# A Statistical Physics View of Pitch Fluctuations in the Classical Music from Bach to Chopin: Evidence for Scaling

**DOI:** 10.1371/journal.pone.0058710

**Published:** 2013-03-27

**Authors:** Lu Liu, Jianrong Wei, Huishu Zhang, Jianhong Xin, Jiping Huang

**Affiliations:** Department of Physics and State Key Laboratory of Surface Physics, Fudan University, Shanghai, China; University of Adelaide, Australia

## Abstract

Because classical music has greatly affected our life and culture in its long history, it has attracted extensive attention from researchers to understand laws behind it. Based on statistical physics, here we use a different method to investigate classical music, namely, by analyzing cumulative distribution functions (CDFs) and autocorrelation functions of pitch fluctuations in compositions. We analyze 1,876 compositions of five representative classical music composers across 164 years from Bach, to Mozart, to Beethoven, to Mendelsohn, and to Chopin. We report that the biggest pitch fluctuations of a composer gradually increase as time evolves from Bach time to Mendelsohn/Chopin time. In particular, for the compositions of a composer, the positive and negative tails of a CDF of pitch fluctuations are distributed not only in power laws (with the scale-free property), but also in symmetry (namely, the probability of a treble following a bass and that of a bass following a treble are basically the same for each composer). The power-law exponent decreases as time elapses. Further, we also calculate the autocorrelation function of the pitch fluctuation. The autocorrelation function shows a power-law distribution for each composer. Especially, the power-law exponents vary with the composers, indicating their different levels of long-range correlation of notes. This work not only suggests a way to understand and develop music from a viewpoint of statistical physics, but also enriches the realm of traditional statistical physics by analyzing music.

## Introduction

Because music has well accompanied human beings for thousands of years, abundant scientific researches have been done to understand the fascinating power of it. For example, a research group used positron emission tomography to study neural mechanisms underlying intensely pleasant emotional responses to music [1]. Voss (1989) discovered self-affinity fractals in noise and music [2]. Tzanetakis and Cook analyzed timbral texture, rhythmic content and pitch content of audio signals to try to classify musical genres [3]. Clearly, these discoveries are still far from enough for people to fully understand interesting laws behind music.

In this work, we attempt to understand music from a statistical physics point of view. Traditional statistical physics mainly concerns about natural systems, whose structural units are usually molecules or atoms. Those units are not adaptive to the environment because they have no mental faculties. From the 1990s, people gradually applied the methods originating from traditional statistical physics to investigate the intelligent and adaptive human systems. For example, Mantegna and Stanley discovered a scaling behaviour of probability distribution for a particular economic index in 1995 [4]. The competing and collaborating activities in a complex adaptive system were also studied to investigate risk-return relationships [5] and resource allocations [6] in human society. Besides, methods of statistical physics were also applied to study the birth (death) rate of words, providing an insight into the research on language evolution [7]. In the light of such directions, here we try extending some of these methods to the field of music, especially the study of notes. In fact, a number of related works have been done before. Manaris et al. (2005) applied Zipf's Law to music and studied the distribution of various parameters in music [8]. Liu (2010) constructed networks with notes and edges corresponding to musical notes and found similar properties in all networks from classical music to Chinese pop music [9]. The research group of Levitin (2012) studied the rhythm of classical music. They computed the power spectrum of the rhythm by the multitaper method, and found a 1/f power law in the rhythm spectra, which can classify different musicians according to the predictability [10]. As far as the classical music is concerned, it is an important branch of music originating in Europe around the 11th century. The central norms and standards of western classical music were codified from 1550 to 1900, also known as the common practice period [11]. It contains three periods: the Baroque era, the Classical era and the Romantic era, when a number of outstanding musicians and masterpieces were born [12]. Therefore, for our purpose, we also focus on the compositions and musicians in this common practice period in the present work. As we all know, a composition of classical music is actually a time series of notes. The time series of pitch fluctuations of notes in a composition correspond to types of melodies, which can distinguish various musical genres and composers. Accordingly, in this work, we mainly calculate the cumulative distribution function (CDF) and the autocorrelation function of pitch fluctuations.

## Methods

We analyze 1,876 compositions of five classical music composers across 164 years [11], [12]. The five composers, including J. S. Bach, W. A. Mozart, L. van Beethoven, F. Mendelsohn, and F. F. Chopin, are the representative figures of three different genres in chronological order, namely the baroque (1600–1750), classical period (1730–1820) as well as the romantic era (1815–1910) [11], [13], [14], [15], [16]. The information of the musicians and the accurate number of compositions we selected are listed in Table 1.

**Table 1:** The information of composers and their compositions.

Composer	Common practice period	Compositions analyzed	Total compositions
Bach (1685–1750)	Baroque music	1114	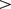 1200
Mozart (1756–1791)	Classical period music	504	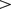 780
Beethoven (1770–1827)	Classical period music	188	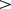 300
Mendelsohn (1809–1847)	Romantic era music	52	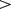 180
Chopin (1810–1849)	Romantic era music	88	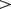 120

All pieces of music in our work were downloaded from kern humdrum music data base [17] as MIDI files, which contain accurate and easily-read information of music. A note in a music score can be named by a scientific pitch notation with a letter-name and a number identifying the pitch's octave [18]. Each scientific pitch notation is corresponding to a certain frequency. Details can be found in Table 2, where the left column (i. e., C, D, E, F, G, A, B) is the note's letter-name and the first line (namely, 0, 1, 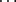, 9) is the pitch's octave. To proceed, we regard the sequential notes or pitches (representing frequencies) of a composition as a time series.

**Table 2:** Frequencies (Hz) of notes, each named by a scientific pitch notation with a letter-name and a number identifying the pitch's octave.

Note	0	1	2	3	4	5	6	7	8	9
**C**	16.352	32.703	65.406	130.81	261.63	523.25	1046.5	2093	4186	8372
**D**	18.354	36.708	73.416	146.83	293.66	587.33	1174.7	2349.3	4698.6	9397.3
**E**	20.602	41.203	82.407	164.81	329.63	659.26	1318.5	2637	5274	10548
**F**	21.827	43.654	87.307	174.61	349.23	698.46	1396.9	2793.8	5587.7	11175
**G**	24.5	48.999	97.999	196	392	783.99	1568	3136	6271.9	12544
**A**	27.5	55	110	220	440	880	1760	3520	7040	14080
**B**	30.868	61.735	123.47	246.94	493.88	987.77	1975.5	3951.1	7902.1	15804

Each scientific pitch notation corresponds to a certain frequency.

Let us denote the pitch of time 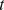 as 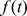 (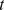 = 1, 2, 3, 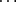, 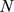), where 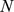 is the length in notes of the concatenated parts of the composition. Then we introduce the pitch fluctuation, 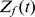, to describe the pitch change between two adjacent notes, which is defined as
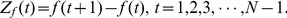
(1)


**Figure 1:**
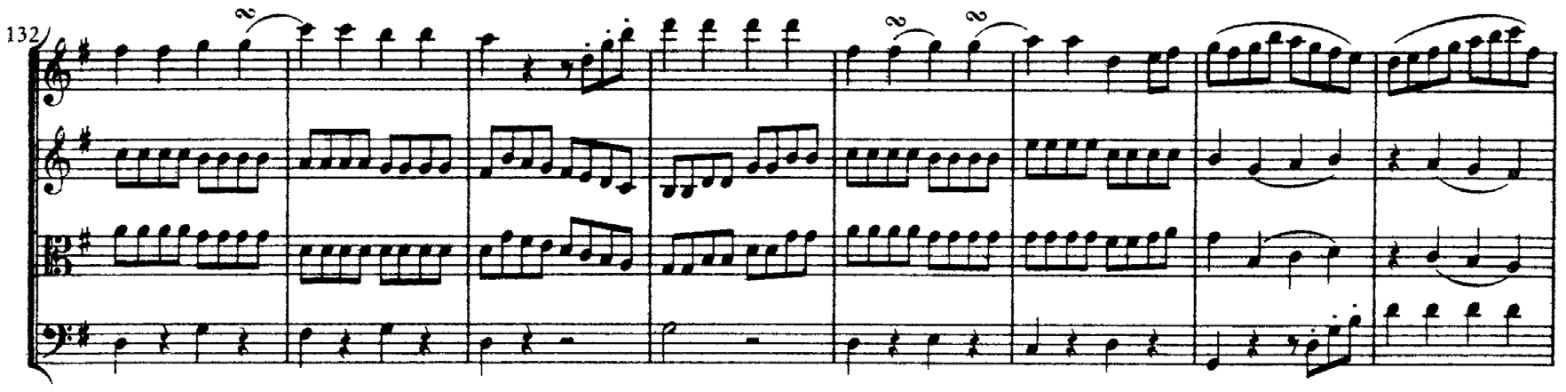
Mozart-Eline Kleine Nachtmusik K.525. Extracted from 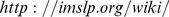.

The reason why we focus on two adjacent notes may be two-folded. Firstly, if we focus on the pitch change between two notes with 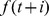 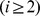 and 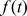, according to Table 2, it can be easily conjectured that the pitch change, 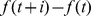, cannot be statistically distinguished well from Bach to Chopin especially when 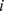 is large enough. Secondly, according to music appreciation, two adjacent notes could be much more impressive for audience than two separated notes with 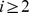. However, it is worth noting that most compositions are composed of several tracks, as shown in Fig. 1. Thus, for our fluctuation calculations, we turn them into one track by adding tracks one after another. Nevertheless, the difference between the ending note of the previous track and the beginning note of the latter track was removed from the calculations throughout this work.

## Results

### (1) Statistical analysis of pitches and pitch fluctuations

**Figure 2:**
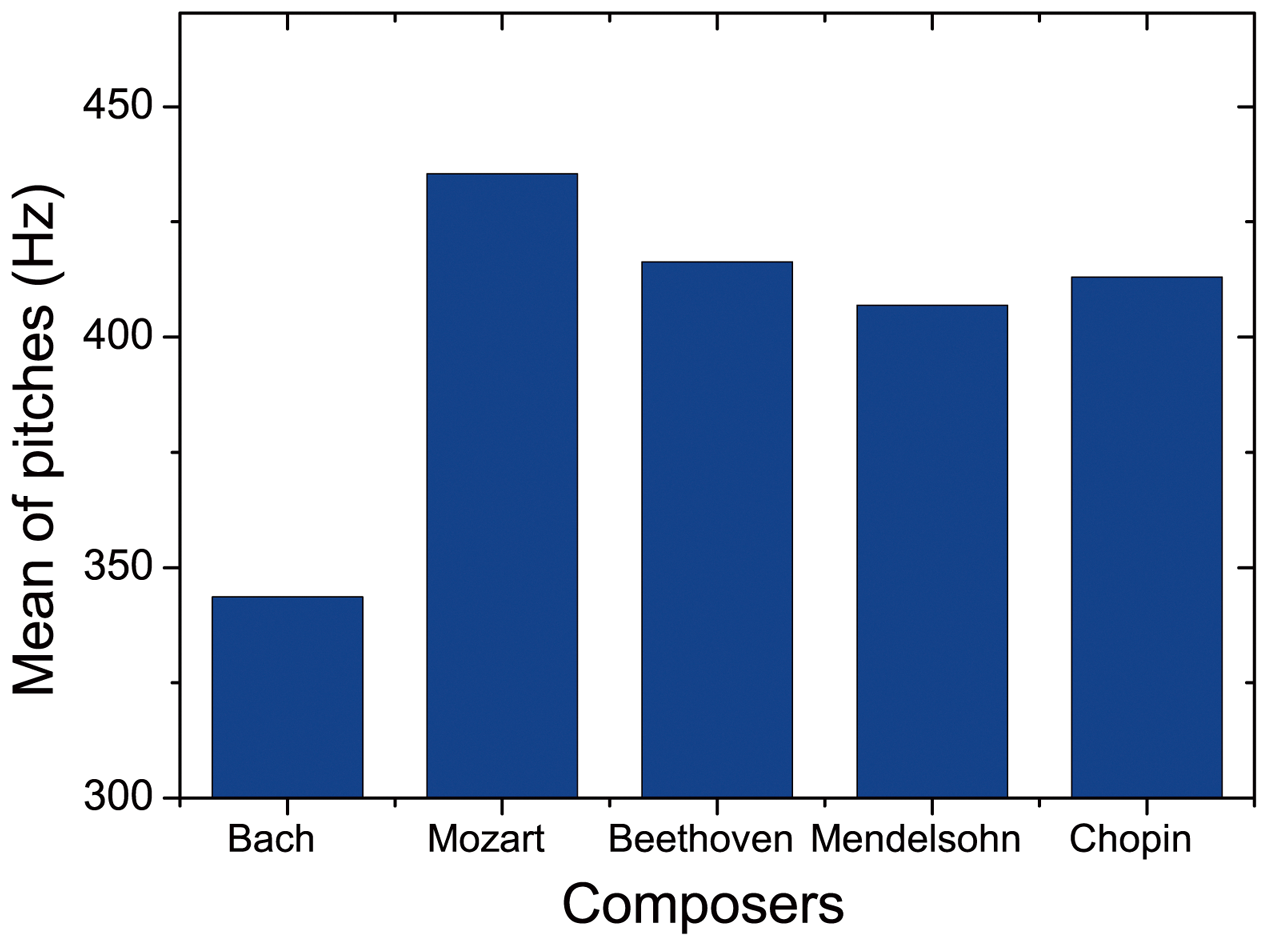
Mean of pitches. The mean value of pitches for the five composers: 343.658 Hz (Bach), 435.448 Hz (Mozart), 416.332 Hz (Beethoven), 406.961 Hz (Mendelsohn), and 314.037 Hz (Chopin).

First, let us take a glimpse at the data of pitches of the five composers, by calculating the mean value of pitches as we can see in Fig. 2. The horizontal ordinate shows the musicians arranged in chronological order according to their years of birth. As we can see, the mean value of pitches is different for the five composers. Particularly, Bach has the smallest value, 343.65 Hz, while the values of the other four composers are all above 400 Hz. In particular, the smallest value for Bach is probably due to the different standards for assigning frequencies in his period, where the tunings were usually lower [19].

Next, let us move on to statistical analysis of pitch fluctuations, 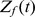. We calculated the mean value and the standard deviation of pitch fluctuations as well as the kurtosis and skewness. All the results are shown in Table 3. As we can see, the mean values of pitch changes are all around zero for the five composers. The kurtosis of Bach is the smallest 8.230 while the kurtosis of Mendelsohn is the largest, 95.953. Speaking of the skewness, Mendelsohn has the value of 1.618 while the values for the rest are much smaller.

**Table 3:** Statistical Analysis of the pitch fluctuations.

Composer	Mean (Hz)	Std. Dev. (Hz)	Kurtosis	Skewness
Bach	−0.361	128.718	8.230	−0.007
Mozart	−0.240	118.987	11.110	0.296
Beethoven	0.784	139.665	16.445	−0.322
Mendelsohn	0.034	158.376	95.953	1.618
Chopin	0.584	159.833	17.689	0.177

After the statistical analysis of pitches and pitch changes, we are now in a position to investigate the CDFs.

### (2) CDF of pitch fluctuations

CDF (cumulative distribution function), 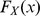, for a discrete variable 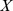 describes the probability distribution of 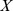 to be found larger than or equal to a number 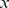 [20], [21]. It is also named as the complementary cumulative distribution function or tail distribution. 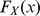 is defined for every number 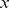 as
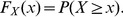
(2)Every CDF is monotonically decreasing. If we define 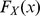 for any positive real number 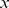, then 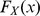 has two properties:
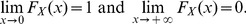
(3)To comply with our notations, here 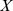 represents pitch fluctuation 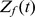. Therefore the positive tail and negative tail of CDF can be calculated separately to make a comparison [22].

**Figure 3:**
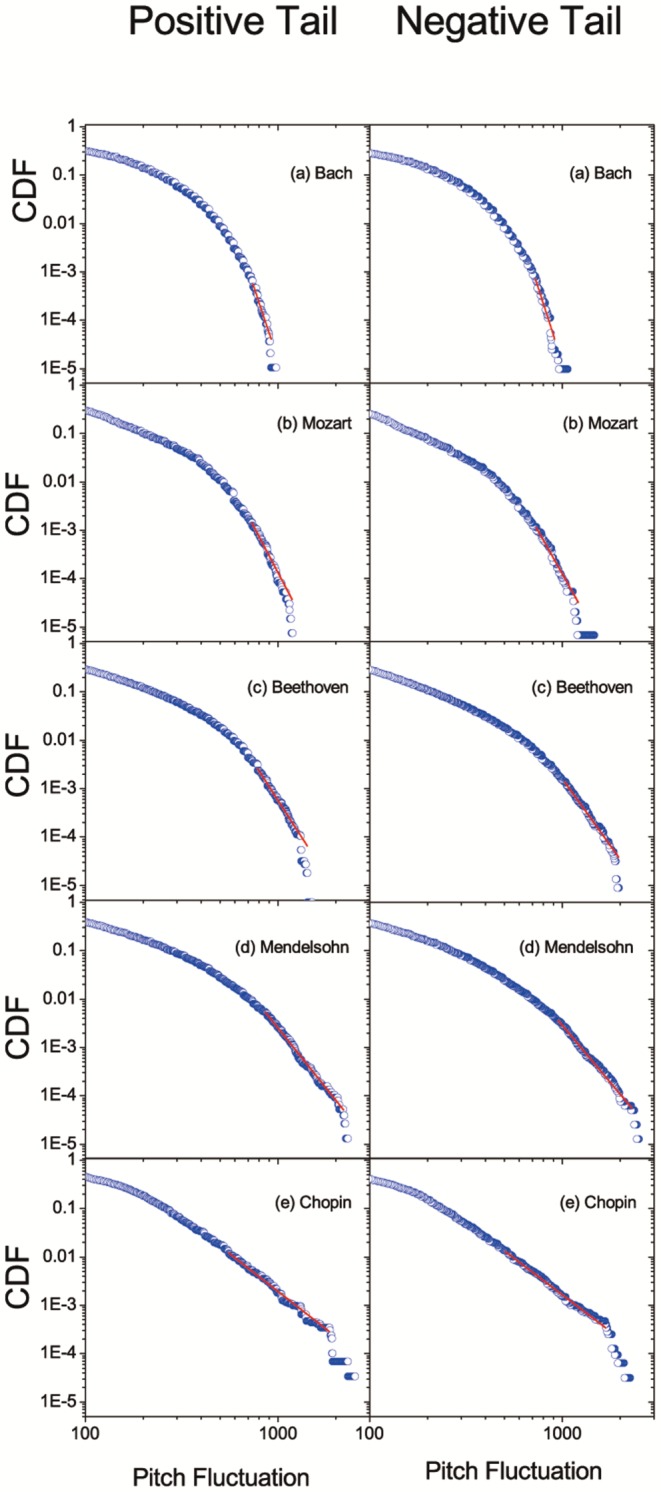
CDF of pitch fluctuations in the log-log plot: (a) the positive tails and (b) the negative tails. All the tails have a part in the power-law (or scale-free) distribution as indicated by the straight lines.

The CDF of pitch fluctuations for each composition is calculated at first, and then it is classified in accordance with musicians, as shown in Fig. 3. Clearly, as time evolves from Bach time to Mendelsohn/Chopin time, the biggest pitch fluctuation of a composer gradually increases. The robustness of this time-evolution result can also be shown because the biggest pitch fluctuations of Mendelsohn and Chopin (born in 1809 and 1810, respectively) are closed very much. Particularly, both positive and negative tails of CDFs show a straight line in the log-log plot for different composers, indicating that the time sequence of the acoustic frequencies, instead of a random process, decays very slowly. Then we applied the power-law fitting to both tails of the CDFs. The fitting formular is
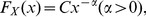
(4)where C is a constant. The corresponding fitting parameters are shown in Table 4. As we can see, each tail of the CDF satisfies a power law, where the power-law exponent 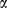 differs from composers. Another discovery is that for the same musician, the positive and negative tails are almost symmetrical except Beethoven, where the 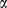 for positive tail is 6.2 and that for negative tail is 5.5.

**Table 4:** The parameters of power-law fits for cumulative distribution functions shown in Fig. 3.

	Positive Tail			Negative Tail		
Composer	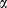	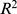	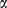 Std. Dev.	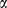	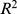	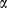 Std. Dev.
Bach	12.059	0.973	0.270	12.396	0.975	0.269
Mozart	8.143	0.979	0.127	7.822	0.978	0.111
Beethoven	6.186	0.992	0.047	5.541	0.985	0.049
Mendelsohn	4.971	0.997	0.015	4.743	0.997	0.014
Chopin	3.11	0.996	0.011	3.021	0.996	0.011

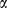
 is the scaling parameter for each composer, 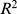 is the regression coefficient and 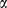 Std. Dev. is the standard deviation for the scaling parameter.

**Figure 4:**
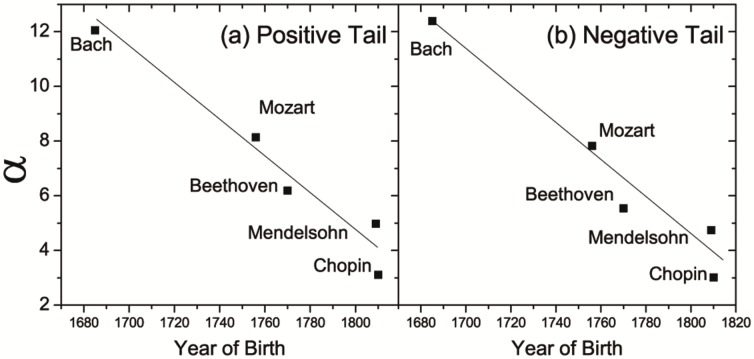
The power-law exponent 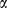 for both the (a) positive and (b) negative tail. 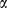
 decreases from Bach to Mendelsohn/Chopin [Note the horizontal coordinates corresponding to the five symbols in either (a) or (b) denote the birth years of the five composers from Bach to Chopin, respectively]. The lines are just a guide to the eye.

Next we examine the time evolution of this scaling property (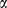), as shown in Fig. 4. The power-law exponent 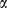 of both the positive and negative tails gradually decreases linearly with time. Because 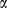 represents the degree of attenuation of the CDF tails, the smaller the exponent is, the slower the tail decays. This reflects that large-scale changes happened more often in the melody. The decay of the tail exponent (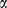) reveals the evolution of classical music that the melody has larger ups and downs from Bach to Mendelsohn/Chopin.

### (3) Autocorrelation function of pitch fluctuations

In statistical physics, the autocorrelation function of a time series describes the correlation with itself as a function of time differences [23]. For a discrete time series, 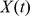, the autocorrelation function, 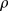, for a time difference, 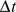, is defined as
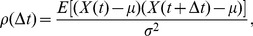
(5)where 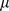 means the mean value of 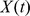, 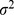 the variance and 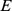 the expected value operator. The value of autocorrelation function changes in range [−1,1], with −1 suggesting perfect anti-correlation and 1 perfect correlation [24]. Here we use 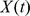 to indicate the absolute value of pitch fluctuations, 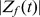.

Different from the calculation of CDF before, we calculate the autocorrelation function of each composition at first, then average the value of autocorrelation of the compositions for each musician. Particularly, we only selected the compositions with more than 250 notes to avoid unusual large values of the autocorrelation functions due to the short length.

**Figure 5:**
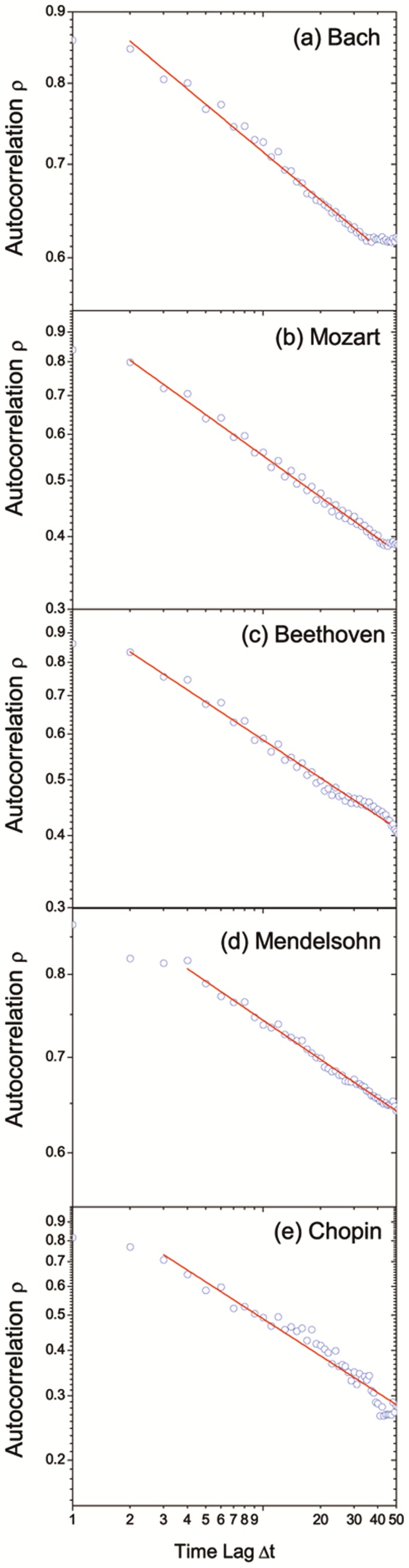
The autocorrelation function 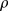 of the absolute values of pitch fluctuations. The horizontal coordinate indicates the time lag, 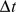, from 1 note to 50 notes, while the vertical coordinate indicates the value of 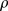. It is worth noting that 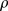 is always positive. In this log-log plot, the five panels respectively show a straight line, suggesting a long-range correlation of notes for each of the five composers.

**Figure 6:**
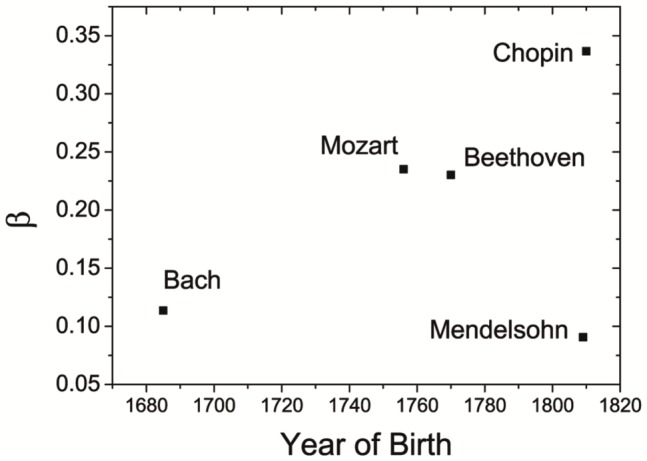
The power-law exponent 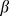 of autocorrelation function. The five composers have different 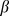
's. Chopin has the smallest value while Mendelsohn has the largest although they were of the same era.

The autocorrelation function for the absolute values of pitch fluctuations is shown in Fig. 5. The values of autocorrelation function for every musician are all positive, which indicate a positive correlation of 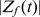. As we can see, the autocorrelation functions for all the five composers in the log-log plot show a straight line (namely, a power-law behavior), indicating a slow decay of autocorrelation functions. Then we applied the power-law fitting to the autocorrelation function. The fitting formular is
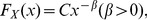
(6)where 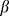 is a constant. The results of power-law fitting are shown in Table 5. As we can see, the power-law exponent (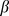) varies with each musician as shown in Fig. 6. This means the decay rate of autocorrelation function is different, or they have different levels of long-range correlation of pitch fluctuations. For example, Mendelsohn has the smallest value of 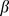 while Chopin the largest.

**Table 5:** The parameters of power-law fits for autocorrelation functions shown in Fig. 5.

Composer	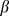	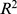	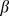 Std. Dev.
Bach	0.114	0.989	0.002
Mozart	0.235	0.992	0.003
Beethoven	0.219	0.986	0.004
Mendelsohn	0.091	0.993	0.001
Chopin	0.337	0.959	0.009

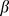
 is the scaling parameter for each composer, 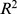 is the regression coefficient and 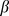 Std. Dev. is the standard deviation for the scaling parameter.

## Conclusions

In conclusion, we have revealed that the biggest pitch change (between two adjacent notes) of a composer gradually increases as time evolves from Bach to Mendelsohn/Chopin. In particular, the positive and negative tails of a CDF (cumulative distribution function) for the compositions of a composer are distributed not only in power laws (i.e., a scale-free distribution), but also in symmetry (namely, the probability of a treble following a bass or that of a bass following a treble are basically the same for each composer). Particularly, the power-law exponent decreases as time elapses. Furthermore, we have also calculated the autocorrelation function of the pitch fluctuations. The autocorrelation function shows a general power-law distribution for each composer. Especially, the power-law exponents vary with the musicians, indicating their different levels of long-range correlation of pitch fluctuations. Compared with the previous works on analyzing music, we focus on pitch fluctuations and study the time evolution and development of the classical music. In particular, all of our statistic results are based on MIDI files. We choose only those five composers due to the limitation of database. However, in the preparation of MIDI files different temperaments, tunings and transpositions in the music were neglected. Works playing with different instruments may correspond to different notes and even form different styles. Thus the statistical results remain to be improved in these aspects. Further, although we study the overall statistical properties of each composer, we should mention that each composer still has various styles in his career and we just have a rough style comparison between composers. This work may be of value not only for suggesting a way to understand and develop music from a statistical physics point of view, but also for enriching the realm of traditional statistical physics by including music.
